# GMO/GMF on Social Media in China: Jagged Landscape of Information Seeking and Sharing Behavior through a Valence View

**DOI:** 10.3390/ijerph16234838

**Published:** 2019-12-02

**Authors:** Rongting Zhou, Dong Wang, Ahmad Nabeel Siddiquei, Muhammad Azfar Anwar, Ali Hammad, Fahad Asmi, Qing Ye, Muhammad Asim Nawaz

**Affiliations:** 1 Department of Science and Technology Communication and Policy, University of Science and Technology of China, Hefei 230026, China; rongting@ustc.edu.cn (R.Z.); wang0817@mail.ustc.edu.cn (D.W.); alihamad564@outlook.com (A.H.); 2 Bond Business School, Bond University, Robina, QLD 4226, Australia; asiddiqu@bond.edu.au; 3 Department of Economic Management, College of information engineering, FuYang Normal University, FuYang 236041, China; 4 Lyallpur Business School, Government College University Faisalabad, Faisalabad 38000, Pakistan; asim5323@yahoo.com

**Keywords:** social media, genetically-modified food, psychological valence, perceived risk, perceived benefits, China

## Abstract

The study examines the critical factors affecting Chinese social media (SM) users’ intentions and behavior to seek and share information on genetically modified organisms/ genetically modified food (GMO/GMF). The proposed framework was conceptualized through benefit-risk analysis and subsequently mapped SM users’ perceived benefits and risks to seeks and share information using Kurt Lewin’s valence view. Quantitative data was collected using survey questionnaires administered from 583 SM users. The results of the path analysis demonstrated two key findings related to SM users’ perceived benefits and risks to seek and share information on GMO/GMF. Among risks, the psychological risk is the strongest predictor of perceived risk to use SM for GMO/GMF, which consequently determines the intentions and behaviors to share information about GMO/GMF on SM in People’s Republic of China. Among benefits, the results showed that perceived usefulness, creditability of GMO/GMF information, and information support are positively related to perceived benefits to use SM for GMO/GMF, which subsequently, predicts the intentions and behaviors to seek information about GMO/GMF on SM. This study suggests scholars and practitioners explore and utilize the efficient communication strategy to fulfill the potential of the SM to increase GMO/GMF acceptance in Chinese society.

## 1. Introduction

The current generation witnessed scientific innovations that revolutionarily reshaped the needs and wants of humanity. For instance, the need to use selective breeding triggered an evolutionary phase of biotechnology in recent decades. This technology profoundly contributes to the rapid scientific breakthroughs in genetic engineering, molecular cloning, and recombinant DNA [1]. These biological applications pulled the discipline to gain considerable attention, particularly the interest and perceptions of scientists and society. Positive constructive communication can be classified as a critical part to unify all critical stakeholders and, consequently, maximize the effectiveness and application of scientific evolution and advancements successfully. In the modern era, social media (SM) has substantially transformed the landscape of communication. Commercial entities are presently using SM to establish positive public relations [1,2] and opinion analysis for value creation [3]. The architecture and design of prominent SM across the globe can be unified to enable individuals to express their opinions and evolve their social ties in the digital sphere [4]. The uniformity of individuals’ opinions, concerns, and intentions glue them together and trigger e-social clusters and events over SM networks, including the creation of pages, groups, live streams, polls, and notifications regarding the virtual events. This nature of e-social events and groups encourages the participants to develop social, psychological, and informational support [5].

In e-society development, SM networks inherit the generalized Internet architecture, which only compels individuals to encounter their desired loads of information. Thus, this feature demands the successful management of socio-scientific issues (SSIs) and related controversies that require active listening, discerning and adopting of a two-way communication strategy to avoid counterarguments [6]. Interestingly, genetically modified food (GMF) is a unique SSI because its outcome and impact are complex and unapparent toward risks and benefits [7,8,9]. This study is primarily conducted to understand the perceived behavior of individuals to measure the potential value gain against the potential risks of sharing and seeking GMF-related information on SM networks. Moreover, this study assumes that this perceived behavior drives the citizen’s actual behavior toward GMF in the People’s Republic of (P.R.) China. Thus, the behavior of individuals toward GMF on SM networks can be gauged over the continuum of benefits and risk. In particular, this analysis comprises Kurt Lewin’s concept of field theory and life-space, which indicates that the valence and movement can be examined and justified; as a result, value of negative and positive forces (velocity) [10]. For instance, the benefit-risk analysis of any commercial and non-commercial product or service exhibits significant effect on consumer behavior, particularly when SSI related issues are involved (i.e., GMF). Furthermore, positive valence can be identified when benefits dominate the risks [9,11,12]. In the next subsection, GMO/GMF-related information over the space of SM in P.R. China is discussed.

### Factual View about GMO/GMF over SM in China

To the best of our knowledge, none of the existing studies have analyzed the Chinese citizens’ usage of SM regarding GMO/GMF through psychological valance view. When the set of studies toward GMO/GMF on Weibo was introduced, the advocates of GMO/GMF adoption observed are Fangzhouzi and Junshi Chen, with a total of 6,141,768 and 523,799 followers, respectively. By contrast, the opinion leaders who hold negative views toward GMO/GMF identified are Yongyuan Cui and Yiwen Chen with a total of 10,314,051 and 252,441 followers, respectively [2]. The trend of massive following toward opinion leaders who oppose GMO/GMF is evident. The SM platforms are utilized by government institutions, academic research firms, media persons, policymakers, lawyers, government institutions, scientists, and citizens to share and seek GMO/GMF-related information. Electronic platforms provide the formal and informal system to communicate efficiently and collaborate to strengthen social ties [3].

**Figure 1:**
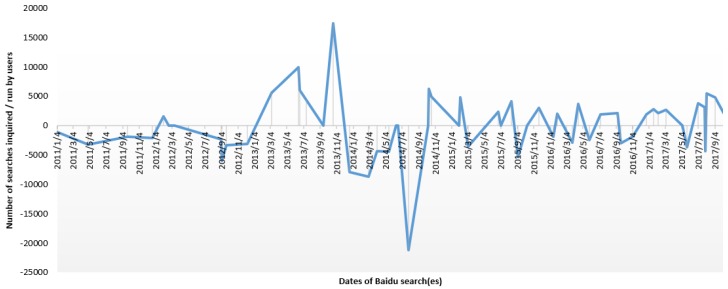
Baidu Search Index (BSI) in case of GMO (x-axis represents timeline as dates are mentioned, Y-axis represents the BSI of each of the event regarding the positive and negative intensity.

**Figure 2:**
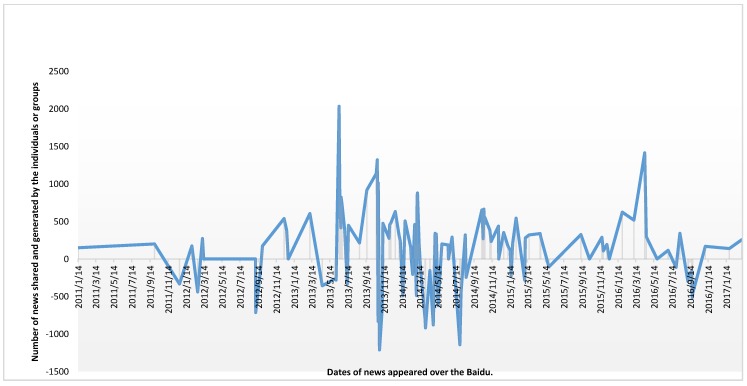
Baidu Media Index (BMI) in the case of GMO (x-axis represents timeline as dates are mentioned, Y-axis represents the BMI of each of the events regarding positive and negative intensity.

In another study, the Baidu search index (BSI) and Baidu media index (BMI) are used to search for the keyword “Genetically Modified” in Chinese (zhuanjiyin), which retrieved 58 and 118 dominating records, respectively. Baidu is China’s leading search engine service over the web. Through keyword searching, Baidu Indexing lists the most related records in graphical and tabular formats based on their relatedness. Baidu Index has been widely used in various practical research, such as forecasting Chinese tourist volume [4], investment trending, and forecasting [5]. Relevant data were obtained during the first quarter of the year 2018 (as a part of problem identification). BSI and BMI were used to depict the “seeking” and “sharing” behavior of users, respectively. BSI highlights the keyword searching trends [6], and BMI represents the volume of shared content of a specific keyword [7]. After manually ranking each of the retrieved records, their negative and positive scores were determined based on the nature of the articles. The results conclude that the trend of negative opinion during 2011–2017 is evident in the BSI. However, BMI indicates that the intensive involvement of government institutions and academics have shifted the direction into a fluctuating opinion that induce controversy. Figure 1 and Figure 2 report the results. The findings from the initial set of observation conclude that SM have stimulated the pace of multidirectional GMO/GMF-related information sharing and seeking of sources presently in the P.R. China. In 2014, Tencent concluded that 90% of the Chinese citizens who participated in the survey exhibit unfavorable opinion toward GMO/GMF [8].

Following the initial study mentioned above, the current study measures the behavior and intentions of individuals toward using SM for sharing and seeking GMF- (as SSI) related information. To rank and include risk- and benefit-related constructs for the current study, intensive literature is reviewed. In particular, to measure the perceived benefits (PBe) (as positive valence) of using SM networks for sharing and seeking information on GMO/GMF, the impact of perceived usefulness (PU) [9], trustworthiness and credibility of information (CI) [10], and information support (IS) [9] are considered. Similarly, to measure the perceived risk (PRi) (as negative valence) to avoid the use of SM sites for sharing and seeking information on GMO/GMF, the constructs of social risk (SoR) [11], psychological risks (PsR) [12], and time risk (TiR) [13] are used. The endogenous constructs from the benefit-risk analysis (interchangeable to valence view) further define intentions and behavior to use SM for seeking and sharing information on GMO/GMF. This study is conducted in the Mainland of China because it possesses a significant segment of the population to reflect the general perception of the country. Besides SM networks, we used Tencent’s, WeChat, and Sina Weiboin the current study. This study’s contributions are notable for the following reasons. First, none of the existing literature has measured the SM users’ behaviors and intentions to share and seek GMO/GMF-related information and developments through the chosen perspective. Second, the behavior toward food technology (GMO/GMF) has never been observed in literature, particularly through valence view. Third, the compound correlation of sharing and seeking intentions in the case of SSI has never been investigated in the hood of independent constructs, as suggested by the current study.

The following sections of the current paper summarize existing academic literature of GMO/GMF-related attitudes among individuals. Moreover, academic research comprising constructs associated with individuals’ habit of sharing and seeking of information is included. The methodology adopted, model-based hypotheses examination, findings, and limitations are also discussed in the subsequent sections.

## 2. Literature Review

The literature on the global social view of GMO/GMF can be categorized into public opinion [14], social representation [15,16], consumer acceptance, production, and safety [17]. Discussion about willingness to pay, willingness to avoid, and willingness to pay to avoid strengthens consumers’’ behavioral mapping in context of agricultural economy [18] and reduces ethical concerns [19]. Interestingly, literature on GMO/GMF in terms of value–risk analysis is comprehensively dominated by the diffused state of micro and macro-level forces [20], such as health risks (allergies, toxicity, and antibiotic resistance) [21], environmental risks (gene transfer and outcrossing) [22], ethical concerns (hunger and intellectual property rights), and nutritious benefits (i.e., golden rice with extra amino acid and yield) with less chemical usage. In summary, the ecological and agricultural risk–value aspects of GMO/GMF have been extensively studied compared with the social aspect, given that the latter is an indirect and philosophical approach for understanding the social view of GMO/GMF. Moreover, examining the social view over the space of regions further complicates the process of monitoring, testing, and predicting trends and behaviors [23].

The SM’s nature of providing a facility to strengthen ties encourages people with similar views to draw together [24]. This similar trend is observable in SSI on SM networks [25]. GMO/GMF subjects on SM networks include social controversies [13], health-related factors [26], and environmental and political factors. In contrast to other SSI factors such as global warming and evolution, GMO/GMF are rarely observed in the existing pool of literature on SM networks. Concerning benefit-risk analysis, GMO/GMF risks are more intensively studied compared with its benefits [27]. Notably, the 1% rule of the Internet culture is entirely dominating in the case of health-related discussions over SM [28]. In particular, the 1% rule of the Internet culture explains that 99% of the users and participants are commonly labeled as lurkers, who merely seek and share information in a less active manner. However, the remaining 1% of the SM users are typically responsible for populating content and participating in discussions [28].

In general, the act of information seeking supports the construction of perceptions on SSI [29]. In most western studies on GMO/GMF, the supportive, positive loads of information can increase trust in technology and reduces the risk perception among the audience [30]. The Internet architecture has complicated information sharing and seeking because of its adoption of pull architecture, which only generates information needs less rationally. On a positive note, the Internet conventionally provides access to comprehensive details [31]. In the case of seeking sensitive information, the Internet facilitates to mask the individual’s identity while inquiring about sensitive aspects [32]. Most of the negative views on GMO/GMF are related to health issues [33]. Neophobia toward GMO/GMF is influenced by culture, experiences, and social traits [34]. Anxiety [35], self-efficacy [36], and literacy [37] are few of the exciting determinants observed for information-seeking behavior. In summary, rare observations regarding benefit-risk analysis on GMO/GMF are previously documented to explain public intentions and behavior to share and seek in the geographical space of the P.R. China [38,39]. The following subsection focuses on the theoretical frameworks and hypotheses of the current study.

## 3. Hypotheses Building

In technological context, benefit-risk analysis argues that the decision to reject or accept any innovation or change is based on the anticipated cost (risk) value analyses performed or perceived image by the individuals [40,41]. In the benefit-risk analysis on GMO/GMF, the transmitters play strongly dominates in amplifying perceived risk (PRi) [42,43]. The current study examines Chinese citizens’ SM usage intentions and behavior to share (USh) and seeks (USe) information on GMO/GMF. Moreover, these intentions and behavior can be used to justify an individual’s choices by his/her input to SM to develop decisions and perceptions on GMO/GMF. The current study comprises perceived benefits (PBe) as the compiled output of the following exogenous factors, namely, the perceived usefulness (PU) of information and discussions on SM networks to understand GMO/GMF, the perceived credibility (CR) of available information (credibility, trustworthiness, and completeness) on GMO/GMF over the SM, and the availability of information support (IS) on SM in case a demand arises when understanding relevant information about GMO/GMF. The other dominating benefits that have been studied are related to different decision-making processes include pricing and affordability [43,44]. The positive value of GMO/GMF can be shared and accessed over social networking sites if the benefits dominate the risk aspects [45]. By contrast, system theory posits that the PRi comprehensively constructs the presumed linkage among micro and macro-level factors. The current study considers the risk aspect rather than safety factors of GMO/GMF, following Luhmann (1979) [45], who argued the ontological difference between safety and risk. Safety is a goal, which deals with uncertainty. Furthermore, safety blocks the decision-making process during a complex and highly uncertain situation, i.e., a citizen’s detailed view of SSI. To integrate PRi in the benefit-risk analysis of Lewin (1939) and Bilkey (1955) [46,47], the determinants from Peter and Tarpey (1975) [48] are considered for the current study. They defined the fear of uncertainty in following dimensions. First, time risk (TiR) refers to the feeling of wasting time while sharing or seeking information on GMO/GMF over SM networks. Second, psychological risk (PsR) serves as a hurdle to obtain beneficial value from GMO/GMF discussion on SM. Third, social risk (SoR) is the PRi that negatively affects an individual’s image and reputation.

### 3.1. Predictors of PBe

PU in technology adoption comprises the end-user’s perception of the innovation to improve job performance [49]. Rogers (1995) [50] emphasized the essence of observability, trialability, and tangibility as significant factors for influencing the intention to adopt and diffuse innovation. The current study assumes that people perceive SM networks as a useful source to effectively understand GMO/GMF. Also, the positive loads of information on SM drive the supportive attitude of people to use GMO/GMF. In particular, the hypothesis is proposed as follows:

**H1(a):**  The PU of GMO/GMF information on SM generates the positive PBe of SM use for GMO/GMF seeking and sharing behaviors.

Trustworthiness, authenticity, and believability are the core to gain trust and credible perception toward GMO/GMF information [51,52]. However, the CI on such a topic has rarely been studied, particularly in terms of social trust and social commerce over the SM space [9]. The study of trustworthiness regarding electronic word-of-mouth has been conducted in the SM sphere while addressing another e-commerce perspective [53], and it has revealed that the credible information pulls end-users of SM to seek and share information. Therefore, this study argues that:

**H1(b):**  *The highly positive CI toward GMO/GMF on SM positively drives the PBe of SM use for GMO/GMF-related information seeking and sharing behavior.*

SM networks strengthen the ties within a community by sharing information, thereby promoting belongingness and support [54,55]. Different socio-scientific concerns and controversies, such as climate change [56], health awareness [57], and GMO/GMF [12], can be observed as top trending threads over the SM space. SM comprises information and knowledge constructs that define the subjective tangibility. Interestingly, the individual’s behavior of sharing and seeking information and knowledge are widely driven by IS as a culture [58]. Thus, in the current study, the SM classified IS as follows:

**H1(c):**  *The positive IS behavior toward SM creates a supportive PBe of SM use for understanding GMO/GMF.*

### 3.2. Predictors of PRi

Neophobia negatively affects the acceptance of GMO/GMF in society because it addresses controversial SSI [33,59,60]. The social cognitive theory discusses that communication is critically dominating the education and psychological clusters of human’s knowledge creation. The weak integrated communication and hardly achievable framing strategy in the case of SSIs (i.e., climate change and GMO/GMF) are transforming the SM space to be complex and skewed where abstractive misconceptions, myths, and phobias are common trends [14]. Thus, the ambiguity in the load of information available on SM triggers the negative perception and peace of mind [61] and increases the magnitude of PRi. Therefore, the current study argues that:

**H2(a):**  *The perceived PsR increases the overall PRi of SM users’ behavior of sharing and seeking information about GMO/GMF.*

In the virtual space, the perceived TiR refers to the doubt and uncertainty to lose time as a valuable resource while rendering information, analyzing opinion, and familiarizing about any observable unit [62]. The controversial SSI-related discussions on SM are commonly end-user driven, wherein different stakeholders express their view and agenda(s) [10,63]. This aspect can occasionally reduce the discussion’s subjectivity and purposiveness. Moreover, the skewness in the opinion can be observed in the discussion of SM; similar trend can be observed in GMO/GMF on SM. Therefore, the current study assumes that:

**H2(b):**  *The perceived TiR can increase the magnitude of overall PRi among SM users who share and seek information on GMO/GMF.*

SoR’s view in the SM sphere deals with the probability of damaging a user’s identity by associating with any irrational argument or controversy [62,64]. The similar nature of SoR has been previously studied while understanding dissatisfaction, regret, and inconsistent intentions to use SM platforms [65]. Thus, the mechanism of how controversial discussions on GMO/GMF [66] relates to the perceived SoR can reduce the user’s engagement on SM. Therefore, the current study proposes that:

**H2(c):**  *The perceived SoR increases the overall PRi of SM users’ information-sharing and seeking behavior toward GMO/GMF.*

### 3.3. Sharing and Seeking Intentions

In the context of diffusion of innovation, people utilize SM networks when the valuable knowledge, social views, and peer reviews on GMO/GMF are communicated with the sense of persuasion (i.e., observability, compatibility and relative advantage) [52,67]. The existing innovation adoption models also emphasize the strong influential power of perceived value to determine the GMO/GMF adoption behavior [9]. In recent years, the seeking and sharing behavior on SM platforms garner considerable attention [68,69]. The PBe of using SM for understanding GMO/GMF can direct the hypothesis and evidence-based search for the sake of seeking and sharing. Therefore, the study proposes the following hypotheses.

**H3(a):**  *The positive PBe of using SM to understand GMO/GMF drives the positive intentions to seek (ISe) information about GMO/GMF on SM.*

**H3(b):**  *The positive PBe of using SM to understand GMO/GMF drives the positive intentions to share (ISh) information about GMO/GMF on SM.*

PRi as a feeling that arises when the end-user anticipates the probability of losing [70]. The acceptance and behavioral studies have concluded that the presence of trust, as a factor, inversely affects risk. In the case of innovation acceptance in society, social trust is necessary when the risk is high. Similarly, institutional trust is observed as a significant dominator on the perceived innovation risk in the existing literature [70,71]. Currently, GMO/GMF discussion becomes increasingly political [72] and skews [66]. Furthermore, the significant part of new public governance [73] and each data source hold different views toward GMO/GMF. Thus, SM networks become highly complicated and confusing when discussing and understanding GMOs. Under the limelight of the existing literature, the following hypotheses are developed:

**H4(a):**  *The increase in PRi of using SM to understand GMO/GMF drives the negative ISe information about GMO/GMF on SM.*

**H4(b):**  *The increase in PRi of using SM to understand GMO/GMF drives the negative ISh information about GMO/GMF on SM.*

### 3.4. Sharing and Seeking Behavior 

The theory of reasoned action, as a behavioral model, argues that the intentions typically exhibit immediate effects on the behavior outcome [74]. The pool of literature on SM adoption exists, which discusses consistent intention based on technological (i.e., anxiety and technostress) [75,76], environmental (i.e., social and peer influences) [77], and task-fit (i.e., compatibility and result orientation) factors [8]. Also, the continuous political and media discussion on GMO/GMF [66], instability in institutional trust [33], and risk from the benefit-risk analysis further explain the user’s willingness and intentions [41]. These components help to extend the current study and provide unique contribution by explaining the following hypotheses:

**H5(a):**  *The positive ISe information about GMO/GMF on SM generates a positive impact on the USe GMO/GMF-related information over the SM.*

**H5(b):**  *The positive ISe information about GMO/GMF on SM generates a positive impact on the USh GMO/GMF-related information over the SM.*

The negative or critical essence of supportive arguments occasionally distracts behavioral intentions. In several instances, rather than seeking, people prefer to share the views to develop a defensive stance or to build a community based on collective view [78]. ISh influences behaviors to share, which is a trend similar to ISe [78]. Interestingly, the information overload on SSI (GMO/GMF) also triggers the sharing behavior, thereby creating a comprehensive view of the SSI (GMO/GMF). In the current study, the second unique contribution of the benefit-risk analysis is demonstrated by the following hypotheses.

**H6(a):**  *The positive ISh information about GMO/GMF on SM generates a positive impact on the USe GMO/GMF-related information on SM.*

**H6(b):**  *The positive ISh information about GMO/GMF on SM generates a positive impact on the USh GMO/GMF-related information on SM.*

## 4. Methodology

An online survey was administered from sample population to achieve the objectives of the study. WeChat and Weibo users in the P.R. China were purposefully selected by using the mix probability approach of convenience and random sampling to understand the target population’s intentions and behavior in the context of benefit-risk analysis on GMO/GMF (Note: Facebook and Twitter were disregarded given that these SM platforms hold limited representation of the Chinese population compared with WeChat and Weibo).

### 4.1. Instruments (Study Scales)

The online survey was intentionally performed to understand the citizens’ intentions to follow GMO/GMF while engaging in the virtual space of the social sphere. In a bifurcated manner, the sharing and seeking intentions to use SM to understand GMO/GMF was investigated. Given that the approach to understanding the sample population was complicated, the existing set scales to measure the instrument were used. These scales have previously been examined and were validated by the current researchers. The item set as measuring scale for PU was observed by Davis (1989) [49], perceived IS by Hajli (2014) [54], perceived CI by Flanagin and Metzger (2008) [79], PBe by Benamati and Rajkumar (2008) [80]. Also, PRi and all of its exogenous factors (PsR, TiR, and SoR) are adapted by Featherman and Pavlou (2003) [81]. The ISh and ISe were observed by Davis and Venkatesh (1996) [82], and the scale to measure USe and USh was observed by following the study of Venkatesh and Bala (2008) [83]. 

### 4.2. Data Collection and Sampling

As a part of the pretest, all researchers contacted a total of 20 undergraduate students who are taking communication- and journalism-related courses. All 20 undergraduate students agreed to review and analyze each of the items for every construct to remove ambiguities and meaningless expressions in the questionnaire. Conceptually, the questionnaire comprised two sections; the first part addressed the demographic characteristics of the sample to sketch the profile of information seekers and sharers of GMO/GMF on SM; the second section addressed the psychological and behavioral view about each construct in the current study to determine the individuals’ behavior toward GMO/GMF on SM. In particular, a seven-point Likert scale that comprised the continuum value from “Extremely Agreed” to “Extremely Disagreed” was used. The informal academic network supported the data collection during the third quarter of 2018 and ensured the confidentiality of the shared information. Moreover, this academic network was also communicated during the follow-up to accelerate the pace of survey collection. The online survey link was sent to nearly 800 SM users. Consequently, a total of 583 SM users responded. Questionnaires with incomplete responses were sorted out and excluded, whereas those with complete responses were considered valid for the current study. These valid questionnaires were further reviewed to avoid nonresponse biases. The researchers reassessed the respondents who previously participated in the survey and found no significant variance between the examined groups. Table 1 presents the demographic profile of the respondents. The criteria for selecting valid representatives of the target population were the respondents who belong to the Chinese consumer market, are active SM users, and are familiar with the GMO/GMF as concept.

The notable trends observed in the collected survey were as follows: First, females dominated the set of respondents (+54%). Second, the age group in the “Quarter Life Crisis” phase is prominent (+55%). Third, the majority of the respondents in the sample population are educated. Fourth and last, nearly all (90%) the respondents are ICT literate cyber-natives, who used to spend not less than an hour over the cyberspace. The demographic profile of the sample depicts the possibility of exploring notable trends while understanding the behavior and intentions of SM users toward GMO/GMF in the digital space. The following section of this paper examines the hypotheses. 

## 5. Data Analysis

To examine the proposed model of the study, the multivariate structural approach, structured equation modeling (SEM), was adopted. SEM is a technique performed by using the SPSS-Analysis of Moment Structures (AMOS) v21 tool by IBM (Armonk, NY, USA), and it allows to examine behavioral and attitudinal models by performing confirmatory factor analysis (CFA), modifications, and fitness indices in the complex multivariate model [84]. 

### 5.1. Measuring Model

During the initial phase of model testing, the exploratory factor analysis (EFA) was performed by using e SPSS Statistics (v.23) (IBM, Armonk, NY, USA) to examine that whether the lower limit of 0.4 was observed in the factor loadings of each item of the constructs [85]. The EFA aims to explore the relationship and structure of the factors and challenge the internal reliability of the factors’ structure [85]. The calculated result from the EFA depicts weak cross-loading scenarios given that the observed values range between 0.892 and 0.990. After the factor loadings analysis, the remaining convergent validity tests, namely, the Cronbach Alpha (CA), the composite reliability (CR), and the average variance extracted (AVE) were performed. The CA generally works to estimate the consistency of psychometric tests and emphasizes the value of inter-item correlation while defining similar construct [85]. The recommended CA value is 0.70 and above. Interestingly, all CAs recorded in the current study are between the range of 0.911 and 0.974. Table 2 presents the results. 

CR deals with the constructs’ items and loadings and the error variance of each item within the construct. AVE deals with the degree of variance hold by the construct verse and measurement error [86]. The recommended threshold value for CR and AVE are 0.70 and 0.50, respectively [85]. Table 2 reveals that all CRs and AVEs recorded in the current study observe satisfactory high values.

In psychometrics, the discriminant (divergence) validity (DV) test confirms the un-relatedness of each construct. Fornell and Larcker suggested that the DV test is performed by comparing each construct AVE’s square root (AVE-SR) value with all the interacting constructs’ correlations. The large AVE-SR value while comparing with inter-construct correlation supports the scale’s validity [85]. Table 3 reports consistent results. To avoid the adverse effect of the constructs, the multi-collinearity was measured by calculating the variance inflation factor (VIF). The VIF recorded was between 1.48 and 2.68 (all less than 10). Therefore, no multi-collinearity issue is present in the current instrument of scale. 

### 5.2. Structural Model

The fitness indices were measured, and the path and CFA were performed during the SEM. Table 4 provides an overview of the model summary and the fitness indices. The observed CMIN as the output of x^2^, and *df* values noted as 3.423 as an output of 1293.755, and 378, respectively. The literature has argued the high sensitivity and dependability of Chi-Square on the sample size [87] and classified that CMIN is acceptable when the observed value is lower than 5.0 [88]. Table 5 indicates that the non-central (CFI and RMSEA), relative (TLI and NFI), and absolute (GFI and AGFI) indices are 0.946 and 0.067, 0.938, and 0.925, and 0.861 and 0.828, respectively, as mentioned in Table 5.

The results from GFI and AGFI observed are lower than the threshold of 0.95 by Hu and Bentler [88]. However, the model finds good fitness based on the previous recommended or accepted values. Table 5 shows all the threshold values. Therefore, the measurement and structural model could be marginally acceptable as supported by the existing pool of literature. Moreover, common method biases were eliminated by reassessing similar respondents through Harman’s one-factor test before the evaluation of hypotheses [85]. Common method biases are present when a single construct can explain half of the variance [85]. In the current study, the construct with the highest value explained 36.10% of the observed variance. Therefore, the present study has non-common common biased method. The following subsection describes the path analysis of each hypothesis.

**Figure 3:**
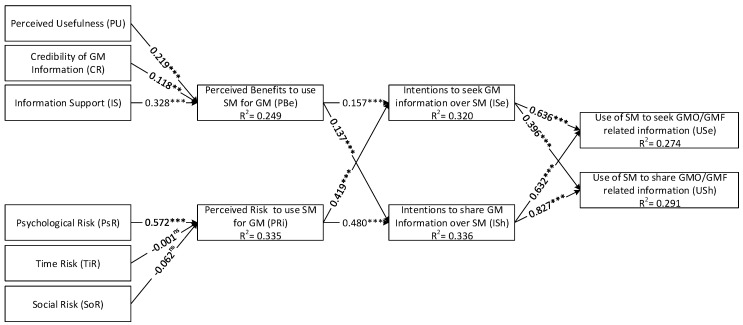
Graphical explanation of path analysis of the proposed model.

Findings reveal that the PBe of using SM for GMO/GMF-related information-seeking and -sharing behavior has significant positive relationship with PU (H1(a): b = 0.219), perceived CR (H1(b): b = 0.118), and perceived IS (H1(c): b = 0.328). Furthermore, the perceived PsT is observed to exhibit a significantly strong relationship with PRi (H2(a): b = 0.527) when using SM for information sharing and to seek on GMO/GMF. However, TiR (H2(b): b = −0.001) and SoR (H2(c): b = −0.062) exhibits nonsignificant and nonsupportive relationship with PRi. The positive PBe helps determine ISe (H3(a): b = 0.157) and ISh (H3(b): b = 0.137). Notably, our study assumes a negative relationship between the PRis of seeking and sharing information through SM and perceived ISh and ISe. However, the tremendously strong supportive association is observed in the case of H4(a):(b = 0.419) and H4(b):(b = 0.418). Table 4 demonstrates these findings. During further examination, the ISe is found to positively influence USe and Ush, given that H5(a):(b = 0.636) and H5(b):(b = 0.396). However, the strong positive Ish is observed as the strongest influencing factor to determine and understand the Ush and Use, given that H6(a) with b = 0.632 and H6(b) with b = 0.827 are recorded. $R^{2}$ is the amount of variance explained by exogenous variables. The Figure 3 demonstrates that the PRi displays high level of predictive power $R^{2}$ given that it explains 33.5% of the variance, whereas PBe explains 24.9%. In addition, ISh (with 33% variance) can better explain the variance compare with ISe (with 32% variance). Similarly, the SM users’ USh (with the 29% variance) dominated the USe (with the 27% variance) when discussing GMO/GMF. Interestingly, the statistical relationship between the control variables observed is non-significant. This finding is further highlighted in the following subsection of this paper.

## 6. Discussion and Conclusions

This study argues the importance of the benefit-risk analysis to define and understand SM users’ perceptions toward GMO/GMF. Through the benefit-risk analysis, we studied the predictability to explain the intentions and behavior of seeking and sharing GMO/GMF-related information on the SM in China. The findings supported the model and provided several interesting relational and statistical results. Thus, the current study marked the valued set of implications.

### 6.1. Theoretical Implications

This study highlighted multiple theoretical implications. First, a lack of previous studies about GMO/GMF understanding of SM applied benefit-risk analysis to classify the information seeking and sharing behaviors of SM users. The previous literature has discussed the opinion toward GMO/GMF in the social context, particularly attitude, demographic, sociographic, and demographic factors. The proposed academic view contributes unique findings to expand the intellectual structure of the knowledge sphere. Second, the scenario of information sharing and seeking about GMO/GMF on SM in China through benefit-risk analysis is depicted, while notable trends are observed compared with the existing non-GMO/GMF benefit-risk analysis-related studies. In particular, in the case of H1, the IS is found as the dominating construct that determines PBe (in contrast to PU and CI). When H2 was examined for conceptualizing PRi, the PsR was found as the dominating factor. However, no significant relationship exists between TiR and SoR in the current study. The rejection of time and social as risks distinguishes the current study among others because it promotes the need to understand ISe and ISh in the context of cognitive and efficacy-based constructs. Third, this study is unique because it is the first initiative to examine ISh and ISe information about GMO/GMF as SSI on SM in China. The findings highlighted the weak positive relationship of H3, which intends to explain the association between PBe, ISh, and ISe. By contrast, in the case of H4, the PRi added value to the study. In particular, the H4 that was initially proposed intended to predict the negative effect of PRi on the ISe and ISh However, a strong positive relationship was found in the current study. Thus, the association between PRi and ISh and ISe highlights the importance of examining the ISe and ISh under customized Chinese cultural constructs, through the spectrum of literacy and science communication/science popularization and fashion. Fourth, the domination of sharing in contrast to seeking in determining intentions and behavior have promoted the current study. Thus, enormous future studies may be conducted to conceptualize the intentions and behavior to seek and share information on GMO/GMF. In particular, when H5 and H6 are examined, the researchers found that ISe has less predictability power compared with ISh to define the USh. However, the exploratory power of intentions to seek and share equally contributed to understanding the USe with slight variation. The current study triggers the immediate need to understand the information evolution about any SSI on SM, particularly when the target audience comes from one of the most populous regions of the world. The intention and behavior to seek and share information about SSI on SM require a holistic view by covering various efficacies, literacies, cognitive abilities, and cultural dynamics.

### 6.2. Practical Implications

Fundamental implications are observed in the current study. At present, the dominating participants on GMO/GMF-related discussion on SM include the government, the media, and the public. In particular, under the government, the officials, scientific researchers of government supervision departments, and scientists from the Chinese Academy of Sciences are directly involved in SM communication. The sphere of the general media comprises commercial science communication media companies (i.e., Guokr, The Intellectual) and non-profit scientific communication organizations composed of scientists and people from all walks of life in media platforms, such as Weibo and WeChat. It emphasizes the future concerns about the source effect and trust in the institutions as critical factors to understand GMO/GMF-related discussion on SM in China. The PsR is a strong determinant that influences PRi but drives the strong intention to seek and share. Moreover, this factor yields supportive behavior. However, the sources of PsR include rumors, government’s failure to supervise GM planting, delay of emergency science popularization, and freedom of voice. For example, rumors that GMO/GMF is fashionable in China to arise. Furthermore, rumors about the false harm of GMF to human and animal health, the false hazard of agriculture and the environment, and inappropriate questions about the nutritional value of GMO/GMF are present. Therefore, to deal with critical circumstances, the Chinese government implemented a legal framework to reduce rumor by adding the law” Amendment IX” to the Criminal Law of the P.R. China on November 1, 2015. The improvement of scientific literacy (SL) can also help the public to identify rumors and deal with SSIs in an intelligent manner. The mechanism to promote immediate science popularization should be highlighted to reduce the negative impact of PsR of GMO/GMF on SM. In the current scenario, the lag of science popularization in the society and on SM has enabled the spread of rumors. Therefore, the strategic use of SM for crisis communication and the public understanding of science and science education can be considered for future studies, thereby creating a supportive and positive effect on GMO/GMF toward acceptance and favorable opinion. The negative effect of restricting the freedom of speech has caused tremendous harm to the government’s image and credibility in the current case of study. A positive attitude can be created by establishing government entities on SM to actively carry out an investigation and public communication in a strategic manner. Consequently, the public is informed, and a two-way hyperactive and constructive communication is developed. The majority of the opinion leaders who support GMO/GMF in the traditional manner and on SM in China attained good science education background. However, owing to the dispersion of influence of their group and ignorance of the principles of communication, they are often under the influence of the public opinion of their opponents. That is, to improve the IS, the institutions should shift from the rational media to a technological advance medium and further communicate the benefits of SM, where the public can obtain confidence and trust in loads of information. 

## 7. Limitations and Future Direction

This study exhibits the following limitations. Firstly, the determinants of benefit-risk analysis were ranked based on the comprehensive review of the literature. However, the socio-cognitive theory, overloads, and cognitive abilities can also be reviewed to understand the SM users’ behavior to seek and share. Secondly, the cross-sectional approach merely constructs public perception and intentions to seek and share about GMO/GMF during the present moment. However, the stakeholders’ dynamics over seven years (2011–2017) have created new interest groups in the SM sphere. Thirdly, the longitudinal study can conclude notable findings based on the research focus. Moreover, the study emphasized the Chinese SM only, which can be potentially examined through the lens of Hofstede’s cultural view, where cross-cultural studies can be conducted, and as the data collected are from the different cities in Anhui Province only. The generalizability of the findings can also be affected. Thus, further studies to validate the study can be conducted.

To emphasize the strategic role of SM, an integrated marketing communication should be developed, where the open and transparent architecture of SM can be used to appreciate the involvement of each critical stakeholder. In addition, citizens should be involved in other SSI related issues, while science communication should be maximized. This study can be extended by involving the critical factors of construct argumentation, informal learning, and understanding science and SSIs. The multiple interdisciplinary views can help obtain fruitful findings by involving the Vision 1 and Vision 2 of SL to communicate about GMF in informal settings (i.e., with the profitable use of SM).

The critical stakeholder management view is a socio-psychological approach to realize the public understanding of science. Therefore, the informal communication strategy that promotes scientific phenomena’s effective and efficient practical implementation for developing a friendly and welcoming society to the science-based interventions. Given that none of the proposed control variables (i.e., age, gender, and education) showed significant effect in the study, purposeful descriptive research can be conducted to underline the relatedness of SSI’s understanding and potential consumers’ demographics. The institutional trust and its dynamics require immediate attention to maximize economic growth and prosperous ecosystem for humankind. The present study focused on the negative aspect, which can create a challenging situation for the future’s science-based innovation in society. This study aims to understand the public’s intentions and behavior on SM while thinking and dealing with SSIs, particularly GMO/GMF. The immediate strategic measures in the most populous region of the world should be assessed for the future generation’s sustainable ecosystem.

## Figures and Tables

**Table 1:** Demographic profile of the respondents.

Profile Parameters		%	Freq.
Gender	Male	45.94	232
	Female	54.06	273
Age group (in years)	Under 18	11.29	57
	18–25	32.87	166
	25–35	43.37	219
	Above 35	12.47	63
Education	Primary/Middle/ Senior High School	37.82	191
	College/University	41.19	208
	Graduate Students	20.99	106
Daily time over SM (in hours per day)	Less than 1	15.25	77
	1–2	30.50	154
	2–3	36.04	182
	More than 3 hours	18.21	92

**Table 2:** Convergent Validity examination of each of the constructs.

Construct	Items	Factor Loadings	Mean	Standard Deviation	CA	AVE	CR
Perceived Usefulness (PU)	PU1	0.955	5.254	1.021	0.925	0.860	0.948
	PU2	0.935					
	PU3	0.892					
Credibility of Information (CR)	CI1	0.966	4.645	1.420	0.974	0.924	0.979
	CI2	0.964					
	CI3	0.962					
	CI4	0.953					
Information Support (IS)	IS1	0.979	5.158	1.467	0.978	0.953	0.984
	IS2	0.975					
	IS3	0.974					
Psychological Risk (PsR)	PsyR1	0.972	4.993	1.227	0.911	0.911	0.954
	PsyR2	0.937					
Time Risk (TiR)	TiR1	0.945	3.964	1.495	0.927	0.869	0.952
	TiR2	0.939					
	TiR3	0.913					
Social Risk (SoR)	SoR1	0.951	4.050	1.877	0.941	0.891	0.961
	SoR2	0.945					
	SoR3	0.935					
Perceived Benefits (PBe)	PB1	0.990	5.042	1.357	0.962	0.958	0.977
	PB2	0.964					
Perceived Risk (PRi)	PRi1	0.954	4.988	1.475	0.945	0.851	0.919
	PRi2	0.890					
Intentions to Seek (ISe)	ISe1	0.977	5.288	1.354	0.968	0.930	0.975
	ISe2	0.958					
	ISe3	0.958					
Intentions to Share (ISh)	ISh1	0.953	4.318	1.594	0.939	0.887	.959
	ISh2	0.941					
	ISh3	0.931					
Behavior to Seek (Use)	BehI1	0.967	5.368	1.374	N/A	N/A	N/A
Behavior to Share (USh)	BehS1	0.974	4.974	1.327	N/A	N/A	N/A

**Table 3:** Descriptive, correlation grid, and AVEs square root value of each of the constructs.

Construct	PU	CR	IS	PsR	TiR	SoR	PBe	PRi	ISe	ISh	Use	USh
PU	0.927											
CR	0.214**	0.961										
IS	0.459**	0.293**	0.976									
PsR	0.427**	0.287**	0.407**	0.945								
TiR	0.082ns	−0.014ns	−0.020	−0.083	0.932							
SoR	0.110*	0.016	0.018	−0.055	0.472**	0.943						
PBe	0.449**	0.460**	0.560**	0.499**	−0.077	−0.099*	0.980					
PRi	0.320**	0.394**	0.308**	0.397**	−0.060	−0.054	0.508**	0.922				
ISe	0.289**	0.305**	0.235**	0.437**	−0.106*	−0.037	0.479**	0.343**	0.964			
ISh	0.133**	0.471**	0.419**	0.340**	−0.096*	−0.028	0.464**	0.348**	0.307**	0.941		
USE	0.452**	0.308**	0.440**	0.487**	−0.062	−0.079	0.581**	0.384**	0.423**	0.304**	1.00	
USh	0.418**	0.407**	0.419**	0.456**	0.014	0.020	0.591**	0.310**	0.335**	0.335**	0.493**	1.00

Note: **p < 0.01; *p < 0.05; ns = not significant, Bold and underline values in diagonal are ‘Square Root of AVEs. PU = Perceived Usefulness, CR = Credibility of Information, IS = Information Support, PsR = Psychological Risk, TiR = Time Risk, SoR = Social Risk, PBe =Perceived Benefits, PRi = Perceived Risk, Ise = Intentions to Seek, ISh = Intentions to Share, USE = Behavior to Seek, USh = Behavior to Share.

**Table 4:** Hypotheses testing and path analysis.

Hypothesis	Description	Significance	Result
H1(a)	PU+ →PBe+	0.219***	Supported
H1(b)	CR+ →PBe+	0.118**	Supported
H1(c)	IS+ →PBe+	0.328***	Supported
H2(a)	PsR+ →PRi+	0.572***	Supported
H2(b)	TiR+ →PRi+	−0.001ns	Not Supported (Statistical)
H2(c)	SoR+ →PRi+	−0.062ns	Not Supported (Statistical)
H3(a)	PBe+ →ISe+	0.157***	Supported
H3(b)	PBe+ →ISh+	0.137***	Supported
H4(a)	PRi+ →ISe-	0.419***	Not Supported (Relational)
H4(b)	PRi+ →ISh-	0.480***	Not Supported (Relational)
H5(a)	ISe+ →USe+	0.636***	Supported
H5(b)	ISe+ →USh+	0.396***	Supported
H6(a)	ISh+ →USe+	0.632***	Supported
H6(b)	ISh+ →USh+	0.827***	Supported

*** *p* < 0.001; ** *p* < 0.01; ns = not significant.

**Table 5:** SEM detailed observations.

Model Fitness Indices	Preferred Degree	Observed Values
CMIN	5.0 [88]	3.423
Chi-Square	Sample size dependent [87]	1293.755
Df	Not specific	378
GFI	0.90 [88]	0.861 (0.86)
AGFI	0.80 [88]	0.828 (0.83)
IFI	0.95 [88]	0.946 (0.95)
TLI	0.95 [88]	0.938 (0.94)
CFI	0.95 [88]	0.946(0.95)
RSMEA	Less than 0.08 [88]	0.069
